# Machine-Learning-Based Laboratory Developed Test for the Diagnosis of Sepsis in High-Risk Patients

**DOI:** 10.3390/diagnostics9010020

**Published:** 2019-02-13

**Authors:** Jacob Calvert, Nicholas Saber, Jana Hoffman, Ritankar Das

**Affiliations:** Dascena, Inc., Oakland, CA 94612, USA; jake@dascena.com (J.C.); nsaber@berkeley.edu (N.S.); ritankar@dascena.com (R.D.)

**Keywords:** sepsis, laboratory developed test, machine learning, clinical decision support, electronic health record, biomarker, medical informatics

## Abstract

Sepsis, a dysregulated host response to infection, is a major health burden in terms of both mortality and cost. The difficulties clinicians face in diagnosing sepsis, alongside the insufficiencies of diagnostic biomarkers, motivate the present study. This work develops a machine-learning-based sepsis diagnostic for a high-risk patient group, using a geographically and institutionally diverse collection of nearly 500,000 patient health records. Using only a minimal set of clinical variables, our diagnostics outperform common severity scoring systems and sepsis biomarkers and benefit from being available immediately upon ordering.

## 1. Introduction

Sepsis, defined as a dysregulated host response to infection [1], is a health crisis affecting over 750,000 Americans annually [2,3], at an estimated cost of over $20 billion per year [3,4]. The mortality rate associated with sepsis is estimated to be as high as one-third [5], and sepsis incidence is estimated to be increasing by more than 10% each year [6]. In response to the challenges clinicians face in treating and diagnosing sepsis, the Surviving Sepsis Campaign has undertaken the improvement and standardization of sepsis treatment [7,8], and procedures have been developed for early sepsis screening, typically using rules-based scoring systems, biomarkers, or combinations thereof [9,10,11].

Rules-based sepsis severity scores, including the Systemic Inflammatory Response Syndrome (SIRS) criteria and the Quick Sequential Organ Failure Assessment (qSOFA) score benefit from their simplicity, transparency, and ease with which they can be calculated [12,13]. However, they have suboptimal sensitivity and specificity in the detection and prediction of sepsis, and have few degrees of freedom for customization to patient populations and sites of implementation. Due in part to advances in patient data availability through the widespread adoption of electronic health records (EHRs) and the making public of de-identified data through databases such as the Multiparameter Intelligent Monitoring in Intensive Care (MIMIC) database [14,15], sepsis detection and prediction using machine learning techniques is now possible, with approaches based on neural nets [16], dynamic Bayesian networks [17], survival-analytic models [18,19], and custom feature engineering [20,21,22,23]. These approaches improve upon traditional rules-based scoring systems by incorporating correlations between clinical variables, nonlinearities, and temporal trends.

These machine-learning-based tools have been developed with the aim of predicting whether or not a patient will develop sepsis in the future or the time of sepsis onset, potentially many hours in advance of onset [18,19]. In this study, we consider instead the problem of diagnosis and develop a machine-learning-based diagnostic (MLD), which is a component of *InSight*—a system for the detection and prediction of sepsis [20,21,22,23]. This laboratory developed test fulfills the role traditionally filled by diagnostic biomarkers. To highlight the benefits of such an approach, we narrow our study to a high-risk group, defined in terms of age and length-of-stay (LOS), for which it would be difficult to develop and specialize a biomarker. The MLD improves upon the diagnostic performance of common scoring systems as well as lactate and procalcitonin (PCT) biomarkers, and further benefits from being developed on a large, heterogeneous dataset, being available immediately upon ordering, and being highly customizable. 

## 2. Materials and Methods 

### 2.1. Datasets for Training and Testing

This study uses a multi-institution, mixed-ward dataset comprised of 489,850 de-identified patient records gathered from the Stanford Medical Center in Stanford, CA, USA; the University of California, San Francisco (UCSF) Medical Center in San Francisco, CA, USA; the Multiparameter Intelligent Monitoring in Intensive Care (MIMIC)-III version 1.3 dataset [15], comprised of patient records from Beth Israel Deaconess Medical Center (BIDMC) in Boston, MA, USA; and the eICU version 1.2 dataset (https://eicu-crd.mit.edu/), representing an assortment of intensive care units (ICUs) across the United States. The Stanford data were collected from all hospital wards from December 2008 to May 2017; the UCSF data consisted of inpatient and outpatient encounters from all hospital wards at the Mount Zion, Mission Bay, and Parnassus Heights medical campuses, all located in San Francisco, CA, USA, collected from June 2011 to March 2016; the BIDMC data consist of ICU stays collected from June 2001 to October 2012; and the eICU data were collected from critical care units at multiple institutions from 2014 to 2015. As we analyzed these data in the context of a diagnostic technology, we are in compliance with Clinical Laboratory Improvement Amendments (CLIA) regulations; the relevant CLIA number for this study is 34D2148112.

### 2.2. Inclusion Criteria

**Figure 1:**
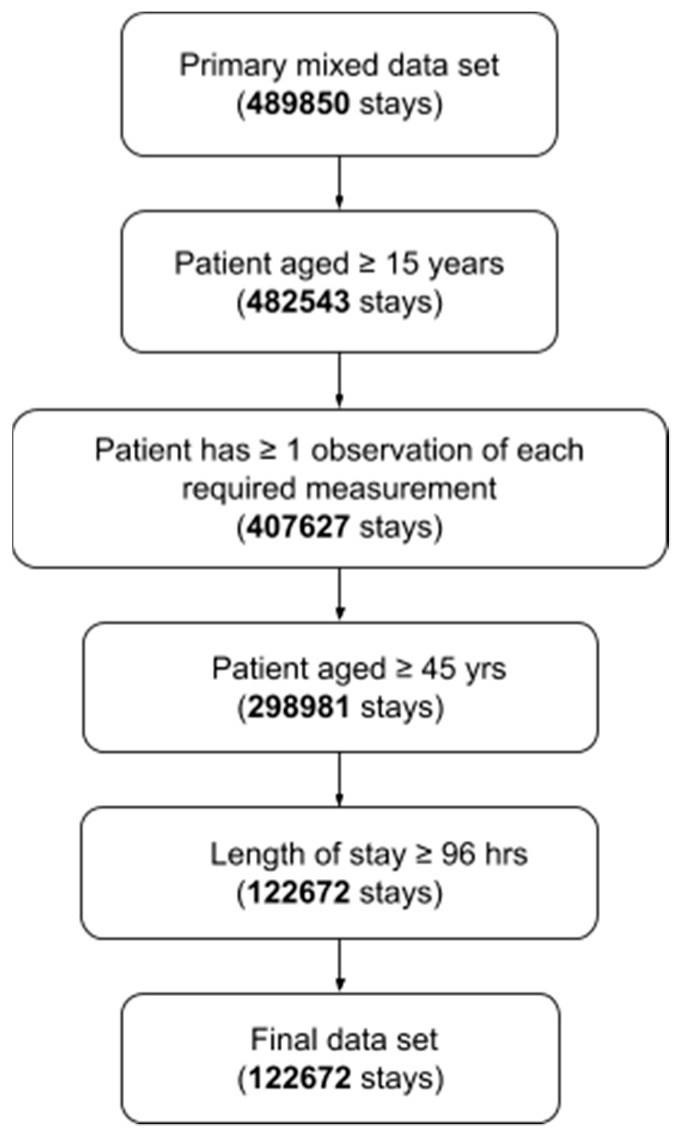
Diagram of inclusion criteria.

Patient stays were subject to inclusion criteria shown in (Figure 1). We defined the high-risk group of patients in terms of age and length-of-stay (LOS). In particular, we examined patients aged 45 years or older and with a LOS of four days or longer. We selected this definition on the basis of data collected from Nacogdoches Memorial Hospital in Nacogdoches, TX, USA from January 2016 to December 2017. Heuristic analysis of the Nacogdoches data indicated that a diagnostic for patients in this group would yield a target rate of expected patients-per-day meeting the definition. We emphasize that, while this definition suffices for our proof-of-concept, other definitions may be chosen to target other high-risk groups or to yield other target rates of patients meeting the criteria.

Patients were required to have at least one observation of each of (i) diastolic blood pressure, (ii) systolic blood pressure, (iii) heart rate, (iv) temperature, (v) respiratory rate, and (vi) peripheral capillary oxygen saturation (SpO2). The required observations could occur at any time during the patients’ stay and were not affected by the LOS cutoff time used to define the high-risk group. Of the 489,850 stays, 122,672 met the inclusion criteria (Figure 1).

### 2.3. Definition of Sepsis Onset Time

We defined the time of sepsis onset in terms of severe sepsis and septic shock standards. Within the high-risk group, for hours at or after the LOS cutoff time, we defined the time of sepsis onset to be the first hour the patient met the severe sepsis criteria or the septic shock criteria, as defined in [20]. For completeness, we include the details here. 

In order for a patient to be labeled as severely septic, their record must include the International Classification of Diseases, Ninth Revision (ICD-9) code 995.92—a diagnostic code indicating the presence of severe sepsis. For severely septic patients, the time of sepsis onset was the first hour in which two or more of the SIRS criteria were met and one of the organ dysfunction criteria were met. The SIRS criteria are (i) heart rate >90 beats per minute; (ii) temperature below 36 °C or above 38 °C; (iii) respiratory rate >20 breaths per minute or partial pressure of carbon dioxide (PaCO_2_) < 32 mmHg; and (iv) white cell count < 4 × 10^9^ cells per L or > 12 × 10^9^ cells per L. 

The organ dysfunction criteria were (i) lactate above 2 mmol per L; (ii) systolic blood pressure below 90 mmHg; (iii) urine output under 0.5 mL per kg, over a 2-h period, prior to organ dysfunction after fluid resuscitation; (iv) creatinine above 2 mg per dL without renal insufficiency or chronic dialysis; (v) bilirubin above 2 mg per dL without having liver disease or cirrhosis; (vi) platelet count below 100,000 µL; (vii) international normalized ratio above 1.5; and (viii) ratio of partial pressure arterial oxygen (PaO_2_) to fraction of inspired oxygen (FiO_2_) under 200, in addition to pneumonia or under 250 with acute kidney injury but without pneumonia.

Finally, we determined the presence of septic shock by the ICD-9 code 785.52, along with (i) systolic blood pressure below 90 mmHg for at least 30 min and (ii) resuscitation with ≥20 mL per kg over a 24-h period; or condition (i) along with (iii) reception of at least 1200 mL in total fluids. For septic shock, the time of onset was defined as the first hour for which (i) and (ii) or (i) and (iii) were met.

### 2.4. Model Diagnostic Problem and Machine Learning Methods

We aim to diagnose sepsis for patients considered at high risk of developing sepsis, due to their age and extended LOS, as soon as they are symptomatic. We fix our definition of sepsis and time of sepsis onset according to the previous section, and allocate patients to the high-risk group. We consider a patient symptomatic if they meet one or more of the SIRS criteria. For example, we imagine a clinician ordering the MLD to diagnose a feverish 56-year-old patient reaching the 8th day of their stay or a 72-year-old patient whose heart rate becomes elevated during the 11th day of their stay. 

After identifying all patients meeting the age and LOS requirements for membership in the high-risk group we determine if the patient meets the criteria for sepsis onset, beginning at the LOS cutoff time and ending at the last hour of the patient’s record. If the patient meets the sepsis definition at or after the LOS cutoff time, we consider the first such time to be the time of sepsis onset. Beginning again at the LOS cutoff time, we identify the first hour that the patient met at least one of the SIRS criteria. In our model scenario it is at this time, which we refer to as the “order time”, that the clinician orders the MLD. Accordingly, we consider as the relevant window of data the three hours preceding the order time, even if the window overlaps with times before the LOS cutoff. It is on the basis of measurements of the required vital signs taken during this window that we make a diagnosis. We emphasize that our goal is limited to the making of a diagnosis, and not the secondary analysis to present a corresponding “explanation” for the diagnosis, on the basis of the features.

Each patients’ time series of measurements was divided into one-hour segments. If a patient had multiple observations of the same clinical variable within the same hour, the measurements were replaced by their average. If a patient had no observation of a clinical variable during a given hour, the most recent observation in the past was carried forward. We used as features the values of the 6 required vital signs during the 3-h window preceding each patients’ order time. We also used the hour-to-hour changes in the vital signs as features. While lab test results were used in the criteria defining sepsis, such data were not used in the training or testing stages. Of course, if such data are available, they may be incorporated into the MLD diagnoses.

We performed 10-fold cross validation by splitting patient records uniformly-at-random into mutually exclusive subsets. We then iteratively joined 8 subsets for training (80%) and used the remaining subset for testing (20%). Our goal was to train and test a gradient-boosted decision trees classifier, using the XGBoost software library [20,24]. The gradient-boosted trees method iteratively trains collections of decision trees to classify the training data; with each step incorporating a new decision tree, which preferentially weights the correct classification of training examples which were previously misclassified. We chose a gradient-boosted decision trees method on the basis of favorable comparison with regression-based methods we have previously studied [21,23], and due to the excellent XGBoost implementation, which provides options for regularization and the handling of imbalanced classes [24]. In addition, we ruled-out naive Bayes classifiers, on the basis of observed dependence between vital signs, when applied to the prediction of sepsis [21].

To prevent overfit of the model to the training data, we included a hyperparameter for the early stopping of the iterative tree-addition procedure. We optimized this hyperparameter for each of the cross-validation folds, without using the 20% testing set, as follows. Within each 80% training set, we uniformly-at-random assigned patients to 1 of 10 subsets of equal size. We then iterated over these subsets, each time training XGBoost on the other 9 of 10 subsets and testing on the remaining subset. For each iteration, we used a different choice of the early-stopping parameter. For each cross-validation fold, we chose the value which led to the best area under the receiver operating characteristic (ROC) curve (AUROC) from the 10 subset test sets. This value was then used for training and testing on the 80% and 20% sets of the respective cross-validation fold. We emphasize that, because the hyperparameter was optimized and selected separately for each fold, no information from the 20% testing set bled into the 80% training during cross-validation.

### 2.5. Comparators

To compare the MLD performance to relevant clinical standards, we applied the SIRS criteria [12], the Modified Early Warning Score (MEWS) [25], and qSOFA [26] to the model diagnostic problem. To obtain diagnoses from these comparators, we calculated their score at the order time. Above fixed score thresholds (e.g., SIRS ≥ 2, MEWS ≥ 5, qSOFA ≥ 2), we considered the comparators as making diagnoses of sepsis. Below their respective thresholds, we considered the comparators as determining the absence of sepsis. We optimized the quality of comparator diagnoses over all possible thresholds. To calculate MEWS, we used the subscoring system of [27]. In particular, we tabulated subscores for heart rate, systolic blood pressure, respiratory rate, temperature, and Glasgow Coma Scale.

We analyzed the test-set performance metrics of the MLD, SIRS, MEWS, and qSOFA, arising from 10-fold cross-validation, as described in the previous subsection. For each fold, we computed a battery of standard metrics for the MLD, and reported the average of each metric across the folds, in comparison with the averages of each metric for the comparators. We chose operating points, the score above which a patient would be considered as given a positive diagnosis and below which the patient is given a negative diagnosis, for the MLD and comparators, which produced sensitivities as near to 0.80 as possible, so as to more easily compare the remaining performance metrics. These choices of operating points do not affect the area under the receiver operating characteristic (AUROC) curve, as it concerns all operating points simultaneously.

## 3. Results

After applying the inclusion criteria of Figure 1, we determined that 18.6% of high-risk patients were septic at or after the 96-h LOS cutoff time. Compared to the SIRS criteria, MEWS, and qSOFA, the MLD performs substantially better at the model diagnostic problem for the high-risk group (Table 1). The MLD obtains an AUROC of 0.917. In contrast, the AUROCs of SIRS, MEWS, and qSOFA were 0.468, 0.639, and 0.653, respectively. We fixed an operating point (corresponding to a particular threshold above which a score was considered as a diagnosis of sepsis) for each score, to produce a sensitivity nearest 0.80 and facilitate comparison with the MLD. For these operating points, we observed a large improvement in MLD positive predictive value (PPV) relative to SIRS, MEWS, and qSOFA. In particular, MLD averaged a PPV of 0.566; the best of the comparators had a PPV of 0.244 (Table 1).

**Figure 2:**
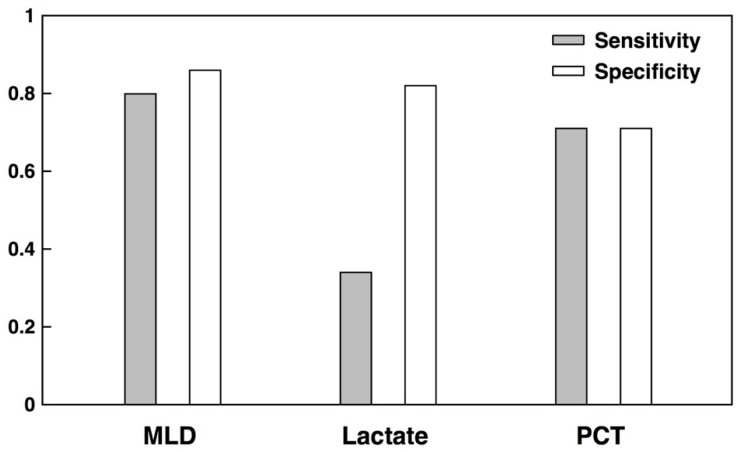
Comparison of average test-set sensitivity and specificity for sepsis diagnoses made on the basis of the machine-learning-based diagnostic (MLD), lactate, and procalcitonin (PCT). The MLD performance is reported as the average over 10-fold cross-validation, for patients aged 45 years or older and with stays exceeding four days. The MLD operating point was chosen to produce a sensitivity near 0.80. Lactate and PCT results are taken from meta-analyses [28,29].

We further compared the MLD at this operating point to the sensitivity and specificity of lactate and procalcitonin (PCT), when used to diagnose sepsis, as reported in meta-analyses [28,29] (Figure 2). The quality of lactate and PCT diagnoses may therefore differ on the patient population we study, for our definition of sepsis, and when used in the context of the model diagnostic problem. Relative to reported performance of lactate and PCT, the MLD has superior sensitivity (0.80 for MLD, compared to 0.34 for lactate and 0.71 for PCT), while improving upon the specificity of PCT (an average of 0.86 for MLD, compared to 0.82 for lactate and 0.71 for PCT).

## 4. Discussion

We developed and tested a machine-learning-based diagnostic for the identification of sepsis in a high-risk group. We chose to define high-risk groups in terms of age and LOS; many alternative high-risk groups are possible. The MLD was developed on a minimalistic set of EHR data, which did not include the outcomes of traditional laboratory tests. While the present study could have included lactate information in the training of the diagnostic, this would have undermined the comparison with reported lactate performance and the availability of lactate results relative to the order time may have required the model diagnostic problem to be altered. The MLD nevertheless outperformed the SIRS criteria and common severity scores at the diagnosis of sepsis (Table 1), in the context of the model diagnostic problem. The MLD compared favorably with the performance of lactate and PCT biomarker, when used to diagnose sepsis, as reported in meta-analyses (Figure 2). These results suggest that the diagnostic may be used in the role traditionally occupied by biomarkers, and offers better performance in terms of sensitivity and specificity.

The MLD offers further benefits over traditional diagnostics. Whereas diagnostics may have a lag period between their ordering and the arrival of results, the MLD’s result is available immediately upon ordering. In fact, we have found, through the prospective implementation of a clinical decision support tooldeveloped using a gradient-boosted trees method, that the time it takes for the trained model to process patient data and produce an alert is on the order of 0.1 s. In the case of a positive test result, this allows clinicians to more quickly take informed action and, in the case of a negative result, prevents the waste of valuable resources, including the unnecessary administration of antibiotics. We emphasize that, because the diagnostic is developed using the most widely available and frequently measured clinical variables, ordering it requires no additional data collection effort from clinical staff. These differences can be associated with improved patient outcomes and reduced costs [30].

We emphasize that, while we do use LOS to identify patients for whom we believe clinicians would like to make a diagnosis, LOS is not incorporated as a variable in the diagnostic model. Were we to deploy such a model in a prospective setting, a patient’s LOS exceeding the four-day threshold would qualify the ordering of an *InSight* diagnosis, but the diagnosis itself would be made on the basis of vital signs, and not the patient’s LOS. For any such patient, a diagnosis would be available immediately—a clinician might order a diagnostic test right away or long after the patient met the criteria for being at high risk of developing sepsis. Moreover, we could similarly develop a diagnostic for a patient population defined differently, such as one which was not defined in terms of LOS. 

In addition to benefits in terms of performance, speed, and costs, the MLD can be developed for patient populations or high-risk groups on which it is impractical to specialize biomarker-based diagnostics. Lactate and PCT, for example, are agnostic of LOS and so cannot use such information without calibrating their diagnostic thresholds accordingly—if at all. Moreover, when biomarker results are available, the MLD can easily incorporate this information to improve its diagnosis. 

Our study suffers from several limitations. The scope of the results is limited to the model diagnostic problem, which may not accurately reflect prospective use. For example, clinicians may not order the appropriate MLD test for high-risk patients during the first hour that they are symptomatic, in which case the model problem differs from the clinical reality. Moreover, the scope of the study did not include the generation of explanations on the basis of the features for each diagnosis. While such explanations may be valuable for the prospective success of such a tool, to provide explanations for each diagnosis requires secondary analysis of the model, which we defer to future work. Our study is further limited by the definitions of sepsis and time of sepsis onset may not capture the entire spectrum of cases for which clinicians would like to diagnose sepsis. This, however, would likely be true of any standard we had chosen, as the latest sepsis definitions [26] have experienced some controversy [31,32]. Finally, we emphasize that our results cannot be claimed to generalize beyond the scope of the patient population under consideration which, while geographically and institutionally heterogeneous, does not represent all patient populations of interest.

## Figures and Tables

**Table 1:** Mean test set AUROC for the Machine Learning Diagnostic (MLD) and comparators when used to diagnose sepsis for patients aged ≥45 years and with stays of ≥96 h. Abbreviations: DOR = diagnostic odds ratio; PPV = positive predictive value; NPV = negative predictive value. To facilitate comparison with the MLD, the diagnosis score thresholds for SIRS, MEWS, and qSOFA were chosen so as to produce a sensitivity nearest 0.80. Performance metrics were averaged over 10-fold cross-validation.

Metric	MLD	SIRS	MEWS	qSOFA
AUROC	0.917	0.468	0.639	0.653
Sensitivity	0.799	0.835	0.774	0.663
Specificity	0.860	0.036	0.317	0.531
PPV	0.566	0.166	0.206	0.244
NPV	0.949	0.489	0.860	0.873
DOR	24.4	0.190	1.59	2.23
Accuracy	0.848	0.185	0.402	0.556
